# Osmotic pressure modulates single cell cycle dynamics inducing reversible growth arrest and reactivation of human metastatic cells

**DOI:** 10.1038/s41598-021-92054-w

**Published:** 2021-06-29

**Authors:** Hubert M. Taïeb, Daniela S. Garske, Jörg Contzen, Manfred Gossen, Luca Bertinetti, Tom Robinson, Amaia Cipitria

**Affiliations:** 1 Department of Biomaterials, Max Planck Institute of Colloids and Interfaces, 14476 Potsdam, Germany; 2 Department of Experimental Neurology, Charité-Universitätsmedizin Berlin, 10117 Berlin, Germany; 3 Institute of Active Polymers, Helmholtz-Zentrum Hereon, 14513 Teltow, Germany; 4 BIH Center for Regenerative Therapies, Berlin Institute of Health at Charité-Universitätsmedizin Berlin, 13353 Berlin, Germany; 5 Department of Theory and Bio-Systems, Max Planck Institute of Colloids and Interfaces, 14476 Potsdam, Germany

**Keywords:** Biological physics, Imaging techniques, Fluorescence imaging, Time-lapse imaging, Cancer microenvironment, Cell growth

## Abstract

Biophysical cues such as osmotic pressure modulate proliferation and growth arrest of bacteria, yeast cells and seeds. In tissues, osmotic regulation takes place through blood and lymphatic capillaries and, at a single cell level, water and osmoregulation play a critical role. However, the effect of osmotic pressure on single cell cycle dynamics remains poorly understood. Here, we investigate the effect of osmotic pressure on single cell cycle dynamics, nuclear growth, proliferation, migration and protein expression, by quantitative time-lapse imaging of single cells genetically modified with fluorescent ubiquitination-based cell cycle indicator 2 (FUCCI2). Single cell data reveals that under hyperosmotic stress, distinct cell subpopulations emerge with impaired nuclear growth, delayed or growth arrested cell cycle and reduced migration. This state is reversible for mild hyperosmotic stress, where cells return to regular cell cycle dynamics, proliferation and migration. Thus, osmotic pressure can modulate the reversible growth arrest and reactivation of human metastatic cells.

## Introduction

The role of the local biophysical microenvironment in cellular function is increasingly acknowledged. Beyond genetically driven intracellular factors, biophysical cues regulate cell metabolism, growth arrest and proliferation^1–3^. It has been shown that compressive mechanical force inhibits spheroid growth due to volume constraints^4^. At a single cell level, mitotic cells in three dimensional (3D) confining microenvironments generate protrusive forces that deform the extracellular matrix, and failure to do so arrests mitosis^5^. For this reason, the deformability and viscoelastic properties of the 3D confining matrices play a key role on cell cycle progression, whereby fast stress relaxation favors cell cycle progression and spheroid growth^6^.

Osmotic pressure is another example of a biophysical cue that modulates cell function. Osmotic pressure changes have been widely associated with growth arrest and proliferation in plants and seeds^7,8^, and more recently in bacteria^9^ and yeast^10^. In human physiology, osmotic regulation is present in multiple phenomena. Tissue architecture results from an equilibrium between forces that expand tissue volume, such as osmotic or hydrostatic fluid pressure, and forces that will counterbalance this expansion, such as tension in the extracellular matrix or contractile forces by cells^11^. Osmotic gradients also arise through the lymphatic system and in blood capillaries in the bone marrow. Specifically, a net positive balance of hydrostatic and osmotic pressure drives fluids out of arterioles, while a negative balance drives fluid back into the venules^12–14^. Interestingly, lymphatic and blood capillary walls are also important cell niches likely to harbor non-cycling quiescent stem cells^15,16^. At a cellular level, osmoregulation is a crucial phenomenon that allows cells to respond to changes in ion concentration in their microenvironment through transmembrane proteins responsible for water/ion transport^17,18^. In a seminal work by Weitz and colleagues, it was shown that variations in external osmotic pressure can induce changes in cell volume, intracellular molecular crowding as a result of water efflux, changes in cell stiffness and ultimately impact mesenchymal stem cell differentiation in the osteogenic or adipogenic lineage^19^.

Yet, the effect of osmotic pressure on single cell cycle dynamics, in particular growth arrest and reactivation, remains poorly understood. Its effect on bulk cell proliferation has been reported, with hyperosmotic pressure resulting in reduced overall proliferation of various human metastatic cells^20,21^. However, less is known about how the osmotic pressure affects single cell cycle dynamics. Quantitative and real-time tracking of the cell cycle dynamics is possible using the fluorescent ubiquitination-based cell cycle indicator 2 (FUCCI2). This tool developed by Miyawaki et al.^22,23^, depicts with fluorescence the different phases of the cell cycle in real-time and is increasingly used to detect non-dividing cells in the context of cancer therapies^24^.

Here, we hypothesize that osmotic pressure can play a role in human cell niches likely to harbor non-cycling quiescent cells by modulating the reversible growth arrest and reactivation into a proliferative state. To test this hypothesis, we use a highly proliferative human metastatic cell line, which we genetically engineered with FUCCI2 to perform quantitative time-lapse imaging of the different phases of the cell cycle at a single cell level. We investigate the effect of different levels of hyperosmotic stress on time-resolved single cell cycle dynamics, nuclear growth, proliferation, migration and protein expression, over a time interval of a few days. Single cell data reveals that under hyperosmotic stress, distinct cell subpopulations emerge with delayed or even growth-arrested cell cycle, impaired nuclear growth and reduced migratory activity. This state is reversible for mild hyperosmotic stress, where cells return to regular cell cycle dynamics, proliferation and migration upon release of the osmotic pressure. These findings on the effect of osmotic pressure on reversible cell growth arrest and reactivation emphasize the importance of the local biophysical microenvironment and have implications in a broader context such as stem cell quiescence vs. proliferation, or human disease like cancer dormancy and metastasis.

## Results

### Hyperosmotic stress slows down cell cycle dynamics of highly and weakly metastatic human breast cancer cells

**Figure 1:**
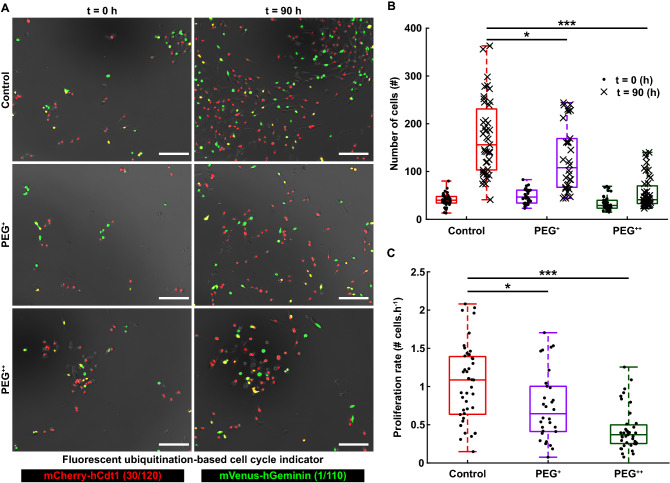
Increase of osmotic pressure slows down the cell cycle dynamics in a concentration-dependent fashion. (**A**) Time-lapse of MDA-FUCCI2 cells exposed to three different osmolalities: 320 mOsm kg^−1^ (control), 380 mOsm kg^−1^ (PEG^+^) and 460 mOsm kg^−1^ (PEG^++^). Scale bars are 200 µm. (**B**) Number of cells at time 0 and after 90 h of imaging (5 biological repeats and N = 43, 30 and 43 number of wells for the control, PEG^+^ and PEG^++^ groups, respectively). (**C**) Proliferation rates are taken as the slopes of the curves indicating the number of cells as a function of time (Supplementary Fig. S1), in the range between 0 and 30 h. The plots represent the median, 1﻿st and 3﻿rd quartiles and extrema. Statistical analysis with respect to the control using a two-tailed Wilcoxon rank sum test, n.s: *p* > 0.05, *: *p* < 0.05, **: *p* < 0.01 and ***: *p* < 0.001.

**Figure 2:**
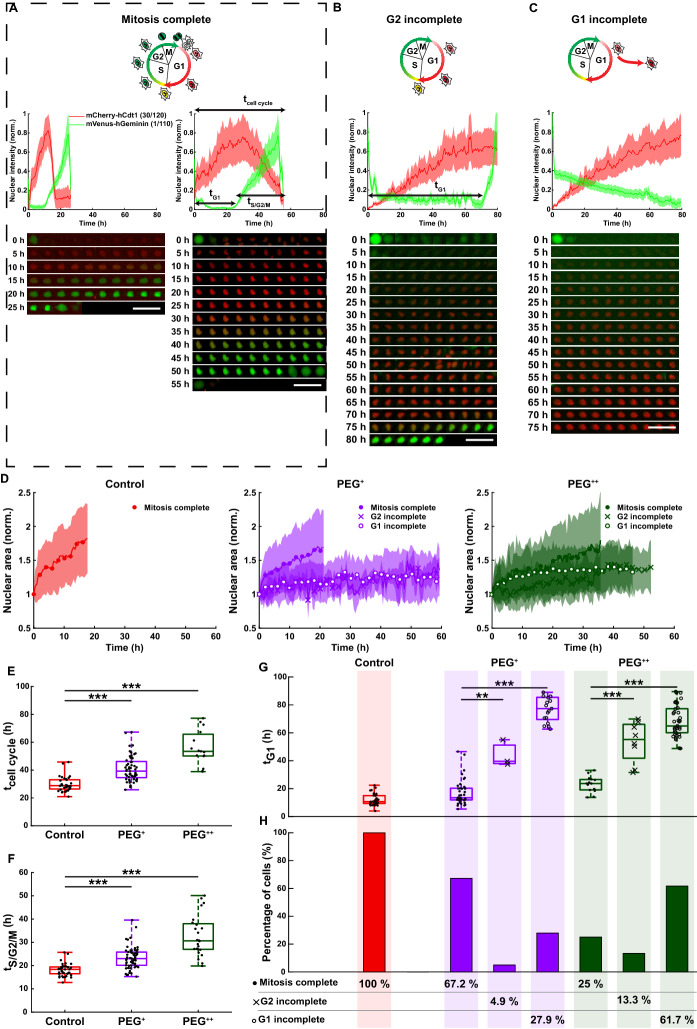
At a single cell level, increase in osmotic pressure leads to the emergence of distinct cell subpopulations with impaired nuclear growth and delayed or arrested cell cycle. (**A**–**C**) Normalized FUCCI2 fluorescence intensity inside of segmented nuclei (middle panels), corresponding fluorescence images over time (bottom panels) and cartoons (top panels) representing the cell cycle phases in three different cell subpopulation: (**A**) cell cycle with mitosis complete (“Mitosis complete”), (**B**) cell cycle with prolonged time in G1 until the cells start the S/G2/M phase (“G2 incomplete”) and (**C**) the cells remain in G1 for the whole duration of the experiment (“G1 incomplete”). The time 0 h for each single cell corresponds to the first frame after division of the parent cell. Scale bars are 100 µm. (**D**) Normalized nuclear area as a function of time for the three experimental groups and the three different cell subpopulations. The line is the average and the shaded area is the standard deviation. (**E**) Duration of the whole cell cycle (time from division to division) for the cell subpopulation “Mitosis complete” (N = 34, 57 and 15 cells for the control (red), PEG^+^ (violet) and PEG^++^ (green) groups). (**F**) Duration of the S/G2/M phase for the cell subpopulation “Mitosis complete” (N = 39, 61 and 24 cells for the control, PEG^+^ and PEG^++^ groups, respectively). (**G**) Duration of the G1 phase in the three different cell subpopulations: “Mitosis complete”, “G2 incomplete” and “G1 incomplete”, for the control, PEG^+^, PEG^++^, groups, respectively. (**H**) Fraction of cells in the three different cell subpopulations, for the groups: control, PEG^+^ and PEG^++^. The plots represent the median, 1st and 3rd quartiles and extrema. Statistical analysis with respect to the control using a two-tailed Wilcoxon rank sum test, n.s: *p* > 0.05, *: *p* < 0.05, **: *p* < 0.01 and ***: *p* < 0.001. The FUCCI2 cartoons were adapted from Sakaue-Sawano et al.^22^, Copyright (2008), with permission from Elsevier.

To understand the role of osmotic pressure in cell proliferation, the highly proliferative human metastatic breast cancer cell line MDA-MB-231 was genetically modified with the FUCCI2 cell cycle reporter (MDA-FUCCI2) and cultured in the presence of 300 Da polyethylene glycol (PEG 300). Osmolality values ranged from control values (320 mOsm kg^−1^, with only cell culture media) to mild hyperosmotic stress (380 mOsm kg^−1^, PEG^+^) and high hyperosmotic stress (460 mOsm kg^−1^, PEG^++^) and the cells were monitored for a period of 90 h every 30 min (Fig. 1A; Supplementary Movie 1). Using the FUCCI2 reporter, the number of cells as a function of time was monitored in real-time. After 90 h, both mild and high conditions significantly impaired proliferation (Fig. 1B) when compared to the control, with a decrease of around four-fold for the highest hyperosmotic stress (PEG^++^). In addition, kinetics of cell proliferation were assessed by calculating the proliferation rate as the slope of the curve: number of cells as a function of time (Supplementary Fig. S1), in the linear range, between 0 and 30 h. The interval of 30 h was determined based on the duration of a total cycle in control condition (Fig. 2E). Notably, already from an early time-point, cell cycle dynamics were highly reduced (Fig. 1C). These finding were reproduced with the weakly metastatic human breast cancer cell line MCF7 stably expressing FUCCI2 (MCF7-FUCCI2) (Supplementary Fig. S4). Namely, that hyperosmotic stress significantly reduced both proliferation (Supplementary Fig. S4B) and cell cycle dynamics (Supplementary Fig. S4C).

To confirm that these results were not specific to PEG, sorbitol was used as an alternative osmolyte, as it is commonly used in osmotic pressure studies^10,25–30^. The same osmolality values were applied over a period of 90 h: control values (320 mOsm kg^−1^, with only cell culture media), mild hyperosmotic stress (380 mOsm kg^−1^, Sorbitol^+^), and high hyperosmotic stress (460 mOsm kg^−1^, Sorbitol^++^) (Supplementary Fig. S5). Analogous to the results obtained with PEG, cell proliferation was significantly impaired when compared to the control (Supplementary Fig. S5B). In addition, the rate of cell proliferation was highly reduced already at an early time point (Supplementary Fig. S5C).

### Single cell analysis of cell cycle dynamics reveals osmotically-driven impaired nuclear growth and delayed or growth arrested cell subpopulations

After the observation that hyperosmotic pressure reduced the overall cell proliferation, we next focused on single cell cycle dynamics of MDA-FUCCI2. Mitosis events were tracked from the beginning of the experiment and up to 30 h. Single cell cycle dynamics were then recorded for the first generation of daughter cells and used in Fig. 2, where time 0 h for each single cell corresponds to the first frame after division of the parent cell. During the G1 phase of the cell cycle, only mCherry fluorescence is expressed in the nucleus as it is linked to the Cdt1 protein, while as soon as the S/G2/M phase starts, the mVenus fluorescence can be detected as it is linked to the Geminin protein (Fig. 2A). Hence, by measuring the fluorescence intensity inside the nucleus over time, the duration of the G1 phase (t_G1_, Fig. 2G), the S/G2/M phase (t_S/G2/M_, Fig. 2F) and the whole cell cycle (t_cell cycle_, Fig. 2E) were evaluated.

**Figure 3:**
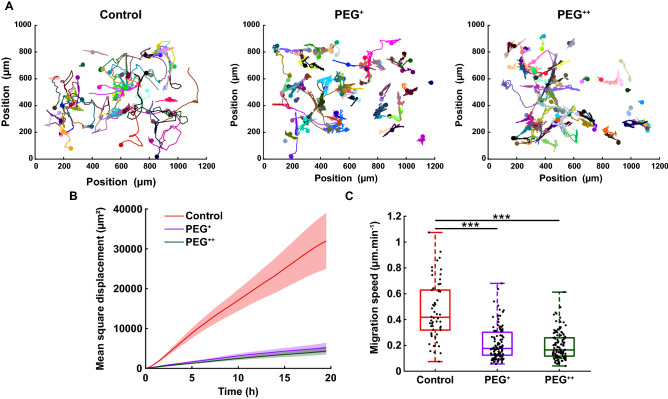
Increase in osmotic pressure strongly reduces cell migration. (**A**) Cell trajectories over the duration of the experiment (90 h), 3 biological repeats and N = 54, 111 and 118 for the control, PEG^+^ and PEG^++^ groups, respectively. Each color represents the path of one cell. (**B**) Mean square displacement of the first 20 h of the cell cycle (MSD, in µm^2^). The thick line represents the average MSD and the shadow is the standard deviation. (**C**) Migration speed (µm min^−1^) of single cells taken as the median of instantaneous cell speeds over their trajectories. The plots represent the median, 1﻿st and 3﻿rd quartiles and extrema. Statistical analysis with respect to the control using a two-tailed Wilcoxon rank sum test, n.s: *p* > 0.05, *: *p* < 0.05, **: *p* < 0.01 and ***: *p* < 0.001.

Under control conditions, all MDA-FUCCI2 cells took 30 h (median) to run through a full cell cycle (Fig. 2E). Interestingly, under hyperosmotic pressure cell cycle dynamics were altered resulting in three different cell subpopulations: (1) the cells could divide but the cell cycle was prolonged (“Mitosis complete”, Fig. 2A right side), (2) the cells had initiated the G2 phase (“G2 incomplete”, Fig. 2B) or (3) the cells stayed arrested in G1 (“G1 incomplete”, Fig. 2C). The duration of the experiment of 90 h corresponds to three times the cell cycle duration under control conditions.

For the cell subpopulation “Mitosis complete”, the osmotically-driven delay in the cell cycle was manifested with an increase in the duration of the whole cell cycle (Fig. 2E), the S/G2/M phase (Fig. 2F) and G1 phase (Fig. 2G, “Mitosis complete”), with a delay proportional to the applied osmotic pressure. All cells of the control group completed mitosis, whereas only 67% and 25% of the mild PEG^+^ and high PEG^++^ hyperosmotic groups respectively managed to do so (Fig. 2H, “Mitosis complete”). Notably, the cell cycle duration could increase up to two-fold between the control and the most osmotically stressed group (PEG^++^).

The cell subpopulation “G2 incomplete” was found in 5% of the cells in the PEG^+^ group and 14% of the cells in the PEG^++^ group (Fig. 2H). This indicates that these cells were highly delayed in their cell cycle progression, as shown by the time spent in G1, with 11 h for the control group, 43 h for the PEG^+^ and 55 h for the PEG^++^ (Fig. 2G, “G2 incomplete”).

Strikingly, a significant fraction of MDA-FUCCI2 cells (28% and 62% for PEG^+^ and PEG^++^ respectively, Fig. 2H) did not even enter the S/G2/M phase (defined as subpopulation “G1 incomplete”) for the whole duration of the experiment (Supplementary Movie 2). Since the cell cycle duration in the control group was 30 h (Fig. 2E), all the cells could have divided three times during the experiment. Instead, the cells in this subpopulation never expressed mVenus-hGeminin (1/110). For this reason, the cells in the subpopulation “G1 incomplete” are considered to be growth-arrested.

Remarkably, for the cell subpopulation “Mitosis complete” in all three experimental groups, the nuclear area increased over time up to roughly two-fold (Fig. 2D, “Mitosis complete”). However, for both cell subpopulation “G2 incomplete” and “G1 incomplete”, under both PEG^+^ and PEG^++^, the nuclear growth was impaired throughout the whole experiment (Fig. 2D, “G1 incomplete” and “G2 incomplete”). Those cells were not dead as they maintained a reduced migratory activity during this prolonged G1 phase (Fig. 3).

### Cell migration is slowed down but not arrested under osmotic pressure

To investigate whether this prolonged cell cycle and reduced proliferation correlated with decreased cell mobility, we examined single cell migration using the FUCCI2 marker. The center of the nucleus of every cell was tracked and their trajectories were recorded over time (Fig. 3A; Supplementary Movie 3). Mean square displacement (MSD) and migration speed were analyzed for the three different groups (control, PEG^+^ and PEG^++^). Under osmotic stress, both MSD and migration speed decreased drastically with respect to the control (Fig. 3B, C). This indicates that the cells under osmotic pressure were less migratory, stayed in a near neighborhood and moved slower than the control group, with median migration speed of 0.42, 0.18 and 0.16 µm min^−1^ for the control, PEG^+^ and PEG^++^, respectively. In addition to reduced proliferation and prolonged cell cycle, cell migration was strongly impaired under hyperosmotic pressure.

### Expression levels of cyclin-dependent kinase inhibitor protein p21 and proliferation marker Ki67 validate the FUCCI2 observations

**Figure 4:**
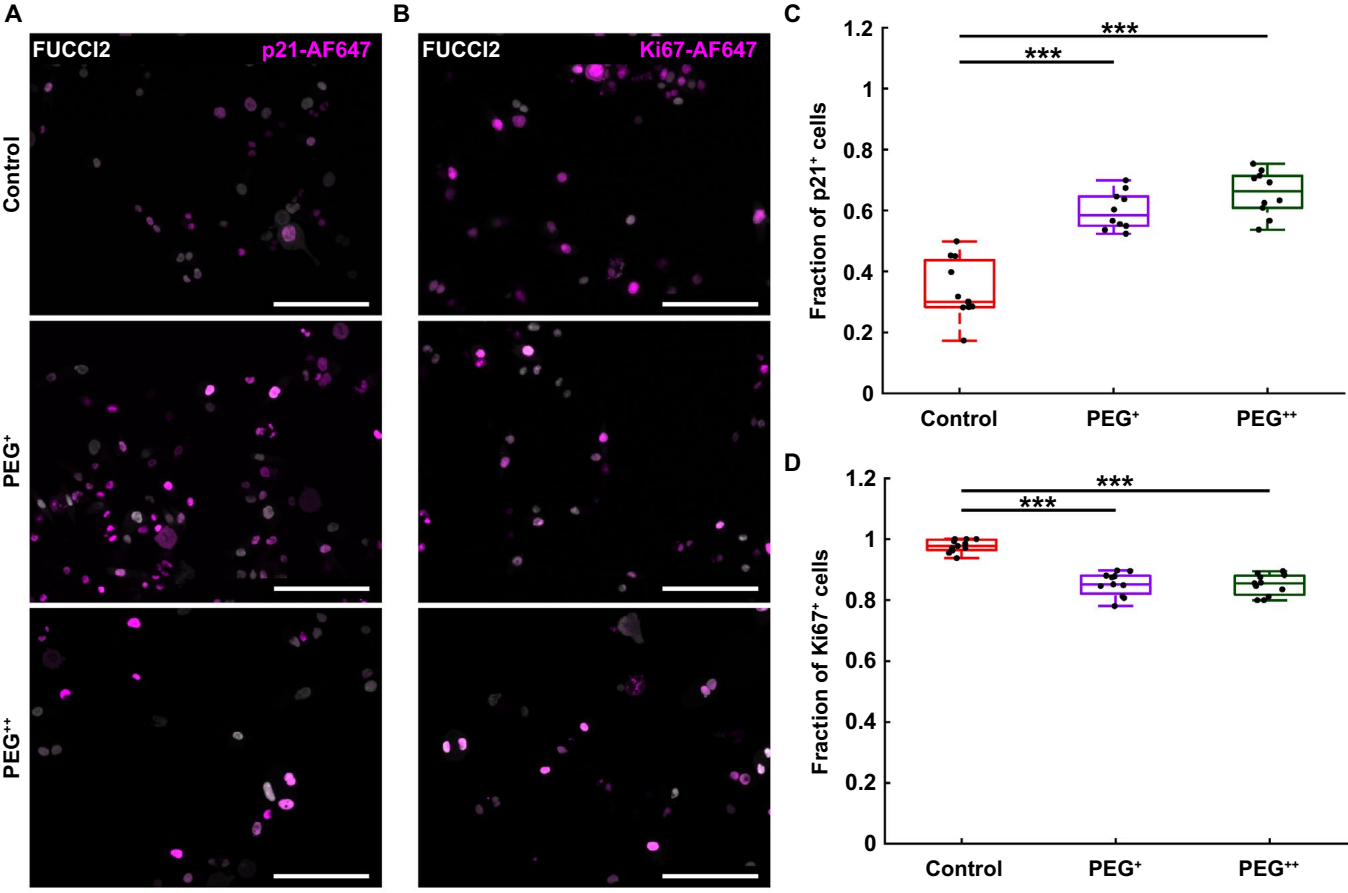
Osmotically-driven delayed or growth-arrested cells show a higher expression of p21 and lower expression of the proliferation marker Ki67. (**A**) p21 immunofluorescence (magenta, AF647) overlaid with FUCCI2 fluorescent cells (artificially indicated as white) for the control, PEG^+^ and PEG^++^ groups, at 90 h time point. Scale bars are 200 µm. (**B**) Ki67 immunofluorescence (magenta, AF647) overlaid with FUCCI2 fluorescent cells (artificially indicated as white) for the control, PEG^+^ and PEG^++^ groups, at 90 h time point. Scale bars are 200 µm. (**C**) Fraction of p21 positive cells on three biological repeats and N = 10 wells. (**D**) Fraction of Ki67 positive cells on three biological repeats and N = 10 wells. The plots represent the median, 1﻿st and 3﻿rd quartiles and extrema. Statistical analysis with respect to the control using a two-tailed Wilcoxon rank sum test, n.s: *p* > 0.05, *: *p* < 0.05, **: *p* < 0.01 and ***: *p* < 0.001.

The FUCCI2 reporter was used to monitor in real-time single cell cycle dynamics under osmotic stress. To further confirm the previous findings describing cell subpopulations with delayed or growth-arrested cell cycle, immunofluorescence staining was performed for the cyclin-dependent kinase inhibitor protein p21 and the proliferation marker Ki67 after 90 h of PEG exposure. Protein expression was detected using a fluorescently labeled secondary antibody (AF467). For clarity, FUCCI2 images were artificially indicated in white and AF647 images in magenta (Fig. 4A, B). Higher levels of osmotic pressure led to a significant increase in the number of p21 positive cells from 30% for the control, to 59% for PEG^+^ and to 66% for the PEG^++^ conditions (Fig. 4C). In accordance with that finding, the number of positive cells for the proliferation marker Ki67 showed a significant drop when increasing osmotic pressure (Fig. 4D).

### Releasing the osmotic pressure reactivates cell proliferation and migration

**Figure 5:**
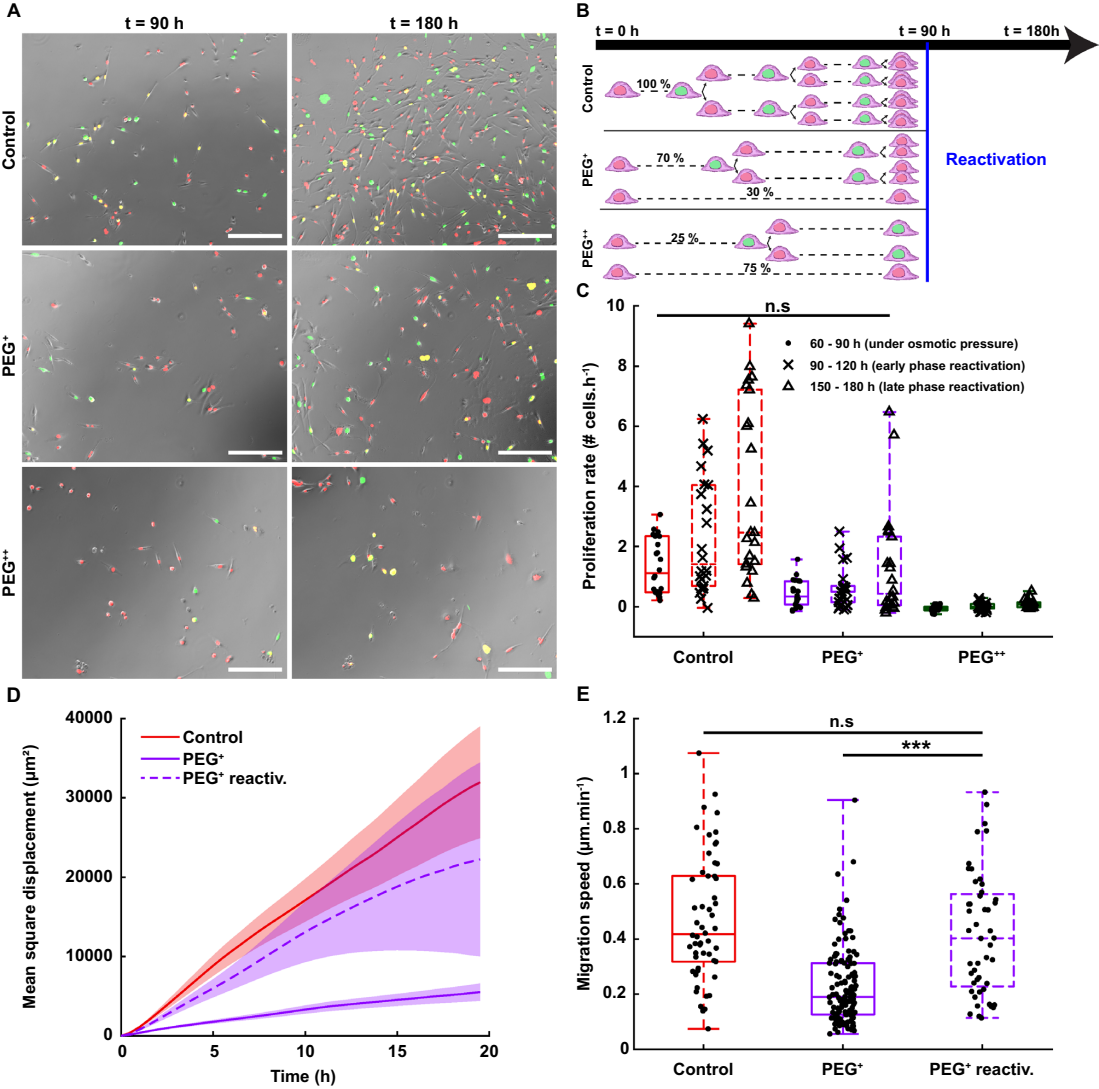
Reversible recovery of cell proliferation and migration, upon return of osmotic pressure to control values. (**A**) Time-lapse of MDA-FUCCI2 cells, previously exposed to 320 mOsm kg^−1^ (control), 380 mOsm kg^−1^ (PEG^+^) and 460 mOsm kg^−1^ (PEG^++^) for 90 h, followed by exposure to control osmolality. Scale bars are 200 µm. (**B**) Illustration of the reactivation experiment pursuing the time-lapse experiment for another 90 h but replacing the cell culture media with standard osmolality (320 mOsm kg^−1^). (**C**) Proliferation rate are taken as the slopes of the curves indicating the number of cells as a function of time in the range between 60 and 90 h (under osmotic pressure), 90–120 h (early phase of reactivation) and 150–180 h (late phase of reactivation), two biological repeats, N = 22 wells for each group. (**D**) Mean square displacement (MSD) of cells that were previously in PEG^+^ (PEG^+^ reactiv., dashed line) in comparison with the previous data for the control and PEG^+^ groups (from Fig. 3B). The thick line represents the average MSD and the shadow the standard deviation. (**E**) Migration speed of cells that were previously in PEG^+^ (PEG^+^ reactiv., dashed box) in comparison with the previous data for the control and PEG^+^ group (from Fig. 3C). The plots represent the median, 1﻿st and 3﻿rd quartiles and extrema. Statistical analysis with respect to PEG^+^ reawk using a two-tailed Wilcoxon rank sum test, n.s: *p* > 0.05, *: *p* < 0.05, **: *p* < 0.01 and ***: *p* < 0.001.

We next investigated whether this delay or arrest of cell cycle, accompanied by a reduction in migratory activity, was a reversible effect. To do so, after 90 h under control, PEG^+^ and PEG^++^ conditions, the media was renewed in all groups with standard cell culture media (Fig. 5B). That is, after 90 h all cells were exposed to the same osmotic pressure as in the control (320 mOsm kg^−1^). The cells were then monitored for another additional 90 h and the same analyses as with the previous experiments were performed (Fig. 5A; Supplementary Movie 4). The proliferation rate was measured as the slope of the curve indicating cell number as a function of time but taken at different times intervals: 60 to 90 h (cells at the end phase of the hyperosmotic pressure experiment), 90 to 120 h (early phase of reactivation), and 150 to 180 h (late phase of reactivation). The proliferation rate of the PEG^+^ group increased over time at the early phase of reactivation (90–120 h) (Fig. 5C, violet) and, at the late phase (150–180 h) it reached values similar to the one of the control group (60–90 h). Cells in the PEG^+^ group also recovered their MSD (Fig. 5D, Supplementary Movie 5), as well as their migration speed (Fig. 5E) and they reached values similar to the control during the first part of the experiment. To our surprise, the cells in the PEG^++^ group did not recover from the osmotic stress and the proliferation rate stayed low even at the late phase of reactivation (Fig. 5C, green).

**Figure 6:**
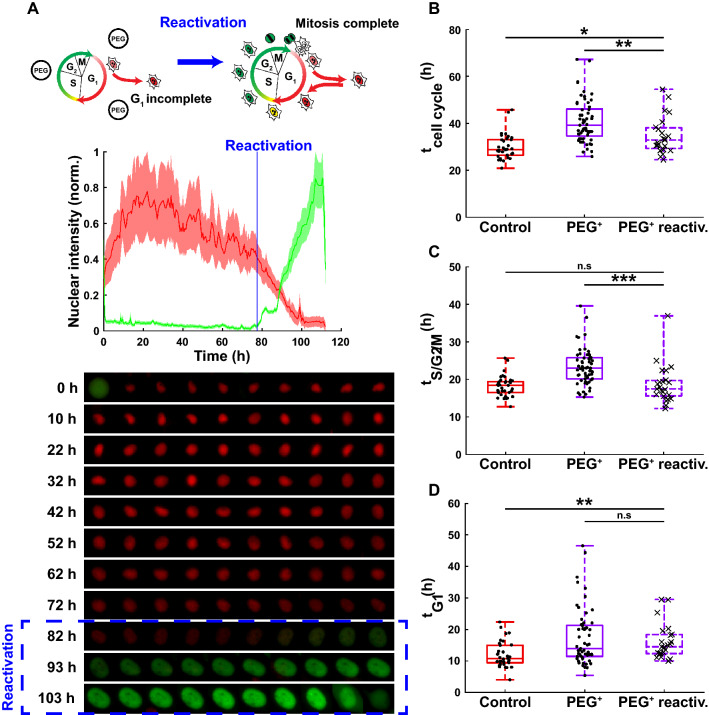
Reactivation and return to regular single cell cycle dynamics of previously delayed or growth arrested human metastatic cells upon releasing the osmotic stress. (**A**) Cartoon of the effect of reactivation on single cell cycle dynamics (top panel), normalized FUCCI2 fluorescence intensity (middle panel), with corresponding fluorescence images before and after reactivation (bottom panel). (**B**) Duration of the whole cell cycle (time from division to division) for the cell subpopulation “Mitosis complete”, before and after reactivation (N = 34, 57 and 23 cells for the control (red), PEG^+^ (violet) and PEG^+^ reactiv. (dashed box), respectively). (**C**) Duration of the S/G2/M phase for the cell subpopulation “Mitosis complete”, before and after reactivation (N = 39, 61 and 23 cells for the control (red), PEG^+^ (violet) and PEG^+^ reactiv. (dashed box) groups, respectively). (**D**) Duration of the G1 phase for the cell subpopulation “Mitosis complete”, before and after reactivation (N = 34, 57 and 23 cells for the control (red), PEG^+^ (violet) and PEG^+^ reactiv. (dashed box) groups, respectively). The plots represent the median, 1﻿st and 3﻿rd quartiles and extrema. Statistical analysis with respect to PEG^+^ reactiv. using a one-tailed Wilcoxon rank sum test, n.s: *p* > 0.05, *: *p* < 0.05, **: *p* < 0.01 and ***: *p* < 0.001. The FUCCI2 cartoons were adapted from Sakaue-Sawano et al.^22^, Copyright (2008), with permission from Elsevier.

In addition to the recovery of properties such as proliferation and migration, single cell cycle dynamics also returned to values resembling control conditions upon reactivation (Fig. 6A). Similar to single cell cycle analyses during osmotic pressure, for reactivation experiments mitosis events were tracked from the beginning of reactivation and up to 30 h. Single cell dynamics were then recorded for the first generation of daughter cells. Interestingly, the duration of the cell cycle after reactivation significantly shortened with respect to the previous hyperosmotic condition and was almost fully returning to control values, with a median value of 33 h for the reactivation group against 30 h for the control (Fig. 6B). The S/G2/M phase after reactivation was also significantly decreased and fully recovered to control values (Fig. 6C). However, the G1 phase duration remained unchanged after reactivation and was therefore still significantly longer than the control (Fig. 6D), although the data dispersion was strongly reduced and resembled the control values.

## Discussion

Together, our findings identify osmotic pressure as a key biophysical cue modulating single cell cycle dynamics that can induce reversible growth arrest and reactivation of human metastatic cells. Quantitative time-lapse imaging of single cell cycle dynamics reveals that, upon exposure to hyperosmotic stress, distinct cell subpopulations emerge with delayed or even growth-arrested cell cycle. This effect is reversible for mild hyperosmotic pressure and cells can return to regular cell cycle dynamics, proliferation and migration upon release of the osmotic pressure. This is in agreement with similar phenomena observed previously with bacteria^9^, yeast cells^29^ and plant seeds^7,8^, but has been barely explored in the context of human cells. Our findings highlight the importance of the local biophysical microenvironment, particularly in human cell niches likely to harbor non-cycling quiescent cells such as lymphatic and blood capillaries in the bone marrow.

We show that hyperosmotic stress drastically impacts highly metastatic MDA-MB-231 cells as well as weakly metastatic MCF7 human breast cancer cells, with a drop in the proliferation rate proportional to the osmotic pressure applied. These results are not specific to PEG and analogous results are obtained when sorbitol is used as osmolyte. This is in agreement with previous observations with prostate cancer cells, where osmotic pressure was linked to a dormant state, but only when seeded at very low (clonogenic) cell density^25^. More recently, Miermont et al.^31^ also observed this drop in proliferation due to hyperosmotic stress in metastatic cell lines, including MDA-MB-231 breast cancer cells, using bulk cell analyses based only on immunofluorescence staining of proliferation markers. Since p53 has previously been related to cell cycle delay or apoptosis^32–35^, the use of a mutant p53 cell line such as MDA-MB-231 could partially explain the reduced proliferation under hyperosmotic stress. However, new experiments performed with the wild-type p53 MCF7-FUCCI2 cell line, and data from the literature with the wild-type p53 T24 cell line^31^, also show a reduced proliferation under hyperosmotic stress. Nam et al.^6^ investigated cell cycle progression under 3D mechanical confinement using hydrogels, with an additional osmotic pressure effect, using static single cell EdU proliferation assays. They found that fast relaxing gels allowed for cell cycle progression, and this was slowed down or arrested for cells within slow relaxing gels, or within fast relaxing gels with an additional osmotic pressure effect. However, to obtain a better understanding of the effects of osmotic pressure on single cell cycle dynamics, a quantitative real-time single cell imaging approach was lacking.

We took advantage of the FUCCI2 reporter to assess single cell cycle dynamics as well as nuclear growth in real-time, with unconfined cells under hyperosmotic conditions and upon release of the osmotic stress. FUCCI2 reporter was developed by Sakaue-Sawano et al.^22,23^ and is increasingly used to study cell cycle progression and especially quiescence^36–40^. Based on single cell analyses, we could distinguish three different cell subpopulations emerging upon exposure to mild (PEG^+^) and high (PEG^++^) osmotic pressure. The subpopulation “Mitosis complete” exhibited prolonged total cell cycle duration (up to two-fold longer than control conditions), with regular nuclear growth. In contrast, the subpopulations “G2 incomplete” and “G1 incomplete” showed impaired nuclear growth, accompanied by prolonged G1 phase for the former (up to five-fold longer than control conditions), and cell cycle arrest in G1 for the latter. The fraction of the subpopulation “Mitosis complete” dropped with increasing osmotic pressure (68% and 25%, for PEG^+^ and PEG^++^), while an increase was observed with higher osmotic pressure in the fraction of the subpopulation “G2 incomplete” (5% and 13%, for PEG^+^ and PEG^++^). Barnet et al.^41^ also observed that the cell populations capable of surviving long-term dormancy were heterogeneous and contained cycle-arrested cells mixed with proliferating cells. The emergence of these different subpopulations of cells under the same biophysical microenvironment highlights the power of single cell analyses in real-time to better understand the dynamics of growth arrest and reactivation of highly proliferative human metastatic cells.

These results were validated with bulk analyses of a collective of cells based on immunofluorescence staining. The osmotic stress resulted in a drop of the proliferation marker Ki67 and an increase of the cyclin dependent kinase inhibitor protein p21 expression, which was proportional to the osmotic pressure applied. It has been shown that p21 expression can be an indicator for cell cycle arrest while inhibiting apoptosis^42^. This cell cycle arrest caused by high p21 expression can happen in both G1 phase, but also in G2 phase by mediation of cyclin B1 degradation in presence of DNA damage^43,44^. In addition, the proliferation-associated protein Ki67 is a common method to visualize growth arrested cells^45–47^. Our immunofluorescence results show that 80% of cells under mild osmotic pressure are Ki67 positive, while the other 20% are Ki67 negative and therefore not cycling (Fig. 4D). This bulk analysis correlates with our findings from the dynamic single cell FUCCI2 analyses in Fig. 2H, with a fraction of 28% cells in G1 incomplete. Both p21 and Ki67 results on bulk analyses agree with the trends detected by summing up the results of the FUCCI2 single cell tracking. Here we quantified a decreasing subpopulation “Mitosis complete” (67% and 25%), increasing subpopulation ‘G2 incomplete” (5% and 13%) and “G1 incomplete” (28% and 62%), from mild (PEG^+^) to high (PEG^++^) osmotic pressure.

Under hyperosmotic stress, cell migration decreased drastically for both mild and high osmotic stress. It has been previously reported that hyperosmotic stress was involved in a reduction or suppression of protrusive activity^48^ which is necessary for cells to migrate^49^. An increase in osmotic stress proved to be responsible for a reduction of lamellipodia area^50^. Conversely, a reduction in osmotic stress, enhanced lamellipodial formation, as shown by an epithelial gap closure assay^51^. These reported effects of osmotic pressure on filopodia and lamellipodia formation could explain our findings of reduced cell migration under hyperosmotic stress.

An essential characteristic of the herein-described cell cycle arrest and reactivation is its reversibility. For the mild hyperosmotic pressure condition, the MDA-FUCCI2 cells resumed proliferation, migration and partially cell cycle dynamics upon releasing the osmotic pressure. Unlike previous studies^25^, this is to the best of our knowledge the first study quantitatively monitoring the reversibility of the cancer cell cycle arrest and return to active proliferation, in real-time and at a single cell level.

One possible mechanism linking osmotic regulation with cell behavior could be the cell and nuclear size. In the context of mesenchymal stem cell biology, it has been shown that variations in external osmotic pressure can induce changes in cell volume, intracellular molecular crowding as a result of water efflux, changes in cell stiffness and ultimately impact mesenchymal stem cell differentiation in the osteogenic or adipogenic lineage^19^. Furthermore, a recent and important study shows that cell division requires the nuclear size-dependent dilution of the cell cycle inhibitor retinoblastoma protein, which is enabled by nuclear growth during the G1 phase^52^. Remarkably, in our study we show that cells exhibiting impaired nuclear growth did not complete mitosis, under both mild and high hyperosmotic pressure; while cells that divided, showed a nuclear growth comparable to control conditions. These findings suggest that a biophysical cue such as osmotic pressure could modulate single cell cycle dynamics by impairing nuclear growth.

While the focus of this study has been the effect of osmotic pressure on single cell cycle dynamics using highly proliferative human metastatic cells as a model system, its implications can be extended to a broader physiological context. Conventional methods such as vapor-pressure depression or freezing-point method are hardly applicable to measure osmotic pressure values in-vivo^53,54^. Recently, a novel biomaterial-based osmotic pressure sensor was developed to possibly fill the gap and enable measurements of osmotic pressure^55^. Osmotic pressure values used in this study were based on those used in previous works^5,19,25,31^, and were below acute hyperosmotic stress values that lead to cell death^56^. It is known that gradients in osmotic pressure and hydrostatic pressure drive fluids out of arterioles and back into the venules^12–14^. Such gradients could contribute to the spatial distribution of quiescent hematopoietic stem cells in the neighborhood of capillaries in the bone marrow^15,16^. From a disease state point of view, growth-arrested human metastatic breast cancer cells have been associated to stable vasculature, while reactivation and proliferation have been linked to sprouting new vessels^57^. Sprouting neovasculature is characterized by a looser and more permeable capillary wall, resulting in a lower osmotic pressure gradient^58^. These findings describing the effect of osmotic pressure on reversible cell growth arrest and reactivation can have implications in a broader context such as stem cell quiescence vs. proliferation, or human disease like cancer dormancy and metastasis.

## Materials and methods

### Lentiviral particle production

Lentiviral vectors mCherry-hCdt1(30/120)/pCSII-EF-MCS (DDBJ/EMBL/GenBank, AB512478) and mVenus-hGeminin(1/100)/pCSII-EF-MCS (DDBJ/EMBL/GenBank, AB512479) were purchased from the Riken Brain Science Institute, Japan (Dr. Atsushi Miyawaki, head of provider laboratory, and Dr. Hiroyuki Miyoshi, developer of pCSII-EF-MCS). Lentiviral particles were generated by co-transfection of HEK-293TN cells (System Biosciences) with mCherry-hCdt1 (30/120)/pCSII-EF-MCS or mVenus-hGeminin (1/100)/pCSII-EF-MCS lentiviral vectors, alongside the packaging plasmid psPAX2 (Addgene plasmid, #12260) and the envelope plasmid pMD2.G (Addgene plasmid, #12259). The culture supernatant was collected and concentrated by ultracentrifugation at 22,000 rpm for 3 h (Beckman L7-55 with SW32Ti rotor) at 4 °C. The virus titer was estimated by transduction on HeLa cells and subsequent flow cytometric analysis for fluorescent protein expression.

### Lentiviral transduction and generation of MDA-FUCCI2 and MCF7-FUCCI2 cells

MDA-MB-231 FUCCI2 (MDA-FUCCI2) and MCF7 FUCCI2 (MCF7-FUCCI2) cells stably expressing FUCCI2 reporters mCherry-hCdt1 (30/120) (red fluorescence) and mVenus-hGeminin (1/110) (green fluorescence) were obtained as follows. Parental cell lines MDA-MB-231 and MCF7 were sequentially infected with lentiviral particles containing mVenus-hGeminin (1/110) at a multiplicity of infection (MOI) of six (MDA-MB-231) or five (MCF7), followed by mCherry-hCdt1 (30/120) at a MOI of three. Successfully transduced cells were identified by expression of mCherry, mVenus or simultaneous mVenus and mCherry fluorescence, sorted with a FACSAria™ II flow cytometer (Becton Dickinson) and expanded for in vitro experiments. Characterization of MDA-FUCCI2 vs. parental cell line MDA-MB-231 was done (Supplementary Fig. S2) to verify that the genetic modification did not significantly change proliferation, migration and adhesion of cells.

### Cell culture

Human metastatic breast cancer cells MDA-MB-231 (ATCC, #HTB-26) and MCF7 (#HTB-22) with the FUCCI2 reporter were cultured in low glucose Dulbecco’s modified eagle’s medium (Sigma, #D6046) supplemented with 1% penicillin/streptomycin (Gibco, #15140-122, 10^4^ units mL^−1^ of penicillin and 10 mg.mL^−1^ of streptomycin) and 10% fetal bovine serum superior (Sigma, #S0615). MCF7-FUCCI2 were additionally supplemented with 0.1% insulin (Sigma, #l2643-50MG). They were grown at 37 °C with 5% CO_2_ on Nunc™ 100 × 17 mm petri dishes (Thermo Fischer, #150350) for regular passaging. The cell line MDA-MB-231 is a mutant p53 cell line, whereas MCF7 a wild-type p53 cell line^59^.

### Osmotic pressure

Increase of osmotic pressure was controlled by adding sterile 300 Da polyethylene glycol (VWR, #8.17002.5000) or D-Sorbitol (Sigma, #240,850) into the cell culture media. Osmolality as a function of PEG 300 concentration was measured with a freezing-point osmometer (Gonotec, Osmomat 3000; Supplementary Fig. S3) to reach the targeted hyperosmotic stress: 380 mOsm kg^−1^ (PEG^+^, 1.5% wt/vol PEG 300) and 460 mOsm kg^−1^ (PEG^++^, 3% wt/vol PEG 300). The same osmolalities values were reached with the D-Sorbitol using 60 mMol L^−1^ (Sorbitol^+^) or 140 mMol L^−1^ (Sorbitol^++^).

### Time-lapse MDA-FUCCI2 experiment

MDA-FUCCI2 cells were washed with phosphate buffered saline (PBS), detached with trypsin (PAN-Biotech, #38220000) and centrifuged at 300 × *g* for 5 min. Cells were then resuspended with cell culture media only (control), or with additional 1.5% wt/vol PEG 300 (380 mOsm kg^−1^, PEG^+^) or with 3% wt/vol PEG 300 (460 mOsm kg^−1^, PEG^++^). They were then seeded at 7500 cells mL^−1^ (200 µL) on a 96 well glass bottom microplate (Greiner Bio-one, #655892) and allowed to equilibrate and adhere for 24 h at 37 °C with 5% CO_2_ before imaging. The cell seeding density was chosen so that the wells remained subconfluent until the end of the imaging period and to reduce possible errors during single cell tracking. After 24 h, cells were placed in a stage top incubator with similar culture conditions (Okolab, UNO-T-H-CO2) mounted on an inverted epifluorescence microscope (Zeiss, AxioObserver 7) for long-term time-lapse imaging. Similar procedure was used with MCF7-FUCCI2 cells.

### Image acquisition

All images were acquired with a Zeiss AxioObserver 7 and a 10x, 0.3 numerical aperture objective (Zeiss, #420341-9911-000). One field of view was taken for each well/condition in the center of the well. Both fluorescence channels mCherry and mVenus were recorded with 100% LED intensity at 511 nm and 555 nm illumination wavelength, respectively. The filter set Zeiss, #46 HE (500/25 excitation and 535/30 emission) was used to image mVenus and the filter set Zeiss, #45 (560/40 excitation and 630/75 emission) to image mCherry, both were recorded at 300 ms exposure. Images were recorded every 30 min for the whole duration of all experiments. The osmotic pressure experiments had a 90 h duration, while the reactivation experiments had a 180 h duration.

### Proliferation assay

Using the time-lapse imaging of MDA-FUCCI2, cell number as a function of time was obtained with a fully automated custom-made MATLAB code as follows. For each frame, both fluorescence channels mCherry and mVenus were independently segmented using an adaptative threshold approach. Then each cluster of pixels, representing each nucleus, was measured in terms of number of pixels. All clusters smaller than 4 µm in radius were discarded. This process was done automatically for all frames to obtain the curves indicating the number of cells as a function of time (Supplementary Fig. S1). Using these curves, the proliferation rate was computed as the slope of a linear fit (y = ax + b, from 0 to 30 h or stated otherwise for the reactivation part).

### Single cell tracking and cell cycle dynamics

Automatic single cell tracking and cell cycle dynamics quantification was done using a custom-made MATLAB application. Mitosis events were tracked from the beginning of the experiment and up to 30 h. Single cell cycle dynamics were then recorded for the first generation of daughter cells, where time 0 h for each single cell (in Figs. 2, 3 and 5) was defined by the first frame after division of the parent cell. This way, the total duration of the cell cycle could be measured from beginning to mitosis. This was necessary as the readout t_G1_ and t_Cell cycle_ are defined with respect to the beginning of the cell cycle. Briefly, the nuclei were automatically segmented, and the nuclear fluorescence intensity as well as nuclear area was being recorded over time. To account for cell variability, the intensity for each channel was independently normalized with respect to its maximum value during the whole cell cycle, for each channel mCherry and mVenus separately. Using the normalized nuclear intensity as a function of time, the duration of cell cycle, G1 and S/G2/M phases were quantified as shown in Fig. 2A (right middle panel). For the cell subpopulation “G1 incomplete” scenario (the S/G2/M phase never started), the t_G1_ was taken as the duration between the start of cell cycle and the end of the experiment (90 h), and was then only a lower limit for the actual duration and not an exact value like for all other cell subpopulations.

### Migration assay

Using the position of the cells from the single cell tracking, cell trajectories were recorded. The mean square displacement (MSD) and instantaneous speed were calculated using the msdanalyzer package in MATLAB^60^. The migration speed was defined as the median of the instantaneous speed over the tracking of the cells.

### Immunofluorescence

After 90 h of imaging, cells were washed with PBS and fixed by adding 200 µL per well of a 4% paraformaldehyde (Boster, #AR1068) solution for 30 min at room temperature. After washing twice with a 3% wt/v bovine serum albumin in PBS, cells were permeabilized with 0.1% wt/v Triton-X-100 (Sigma-Aldrich, #T8787) in PBS for 10 min at room temperature. After two washing cycles, cells were incubated for 17 h in the fridge, either with a 305 ng mL^−1^ p21 Waf1/Cip1 primary antibody (Cell Signaling Technology Europe, #2947S) in a dilution buffer containing PBS + 3% wt/v BSA + 0.1% Triton-X-100, or with a 1.25 µg mL^−1^ Ki67 primary antibody (abcam, #ab15580) in the same dilution buffer, except for the negative controls in each group. After washing twice, all cells were incubated for 1 h at room temperature in 10 µg mL^−1^ Alexa Fluor 647 goat anti-rabbit antibody (Life Technologies, #A21244) in the same dilution buffer. After two washing steps, cells were imaged using the same microscope and settings as described above. A custom-made MATLAB application was used to count the number of p21/Ki67 positive cells. Briefly, the two channels from the FUCCI2 reporter were artificially merged and used to localize the cells. The negative controls of p21 and Ki67 were then used to determine the background signal of the secondary antibody and thereby define the threshold for positive p21 or Ki67 signal.

### Reactivation experiments

After the 90 h initial experiment under the three different conditions (Control, PEG^+^ and PEG^++^), the media was renewed in all groups with standard cell culture media (Fig. 5B). Time-lapse imaging was performed during additional 90 h, at the exact same location. Similar analyses were performed to extract the MSD, migration speed, and single cell cycle dynamics. To do so, mitosis events were tracked from the beginning of the reactivation and up to 30 h. Single cell cycle dynamics were then recorded for the first generation of daughter cells. This way, the cell cycle total duration for each cell could be measured. This defines the time = 0 h in Figs. 5 and 6.

### Statistical analysis

All data were analyzed using MATLAB. The plots represent the median, 1﻿st and 3﻿rd quartiles and extrema. Statistical analysis was done with respect to the control using a non-parametric two-tailed Wilcoxon rank sum test except when stated otherwise, n.s: *p* > 0.05, *: *p* < 0.05, **: *p* < 0.01 and ***: *p* < 0.001.

## Supplementary Information

Supplementary Information 1.

Supplementary Information 2.

Supplementary Information 3.

Supplementary Information 4.

Supplementary Information 5.

Supplementary Information 6.

## Data Availability

Data available in a publicly accessible repository of the Max Planck Society (https://dx.doi.org/10.17617/3.64).
