# Publisher Correction: Osmotic pressure modulates single cell cycle dynamics inducing reversible growth arrest and reactivation of human metastatic cells

**DOI:** 10.1038/s41598-021-99039-9

**Published:** 2021-09-24

**Authors:** Hubert M. Taïeb, Daniela S. Garske, Jörg Contzen, Manfred Gossen, Luca Bertinetti, Tom Robinson, Amaia Cipitria

**Affiliations:** 1 Department of Biomaterials, Max Planck Institute of Colloids and Interfaces, 14476 Potsdam, Germany; 2 Department of Experimental Neurology, Charité-Universitätsmedizin Berlin, 10117 Berlin, Germany; 3 Institute of Active Polymers, Helmholtz-Zentrum Hereon, 14513 Teltow, Germany; 4 BIH Center for Regenerative Therapies, Berlin Institute of Health at Charité-Universitätsmedizin Berlin, 13353 Berlin, Germany; 5 Department of Theory and Bio-Systems, Max Planck Institute of Colloids and Interfaces, 14476 Potsdam, Germany

Correction to: *Scientific Reports* https://doi.org/10.1038/s41598-021-92054-w, published online 29 June 2021

The original version of this Article contained typographical errors.

In Figure 2, the text was incorrectly displayed in small caps. The original Figure 2 and accompanying legend appear below.

**Figure 2:**
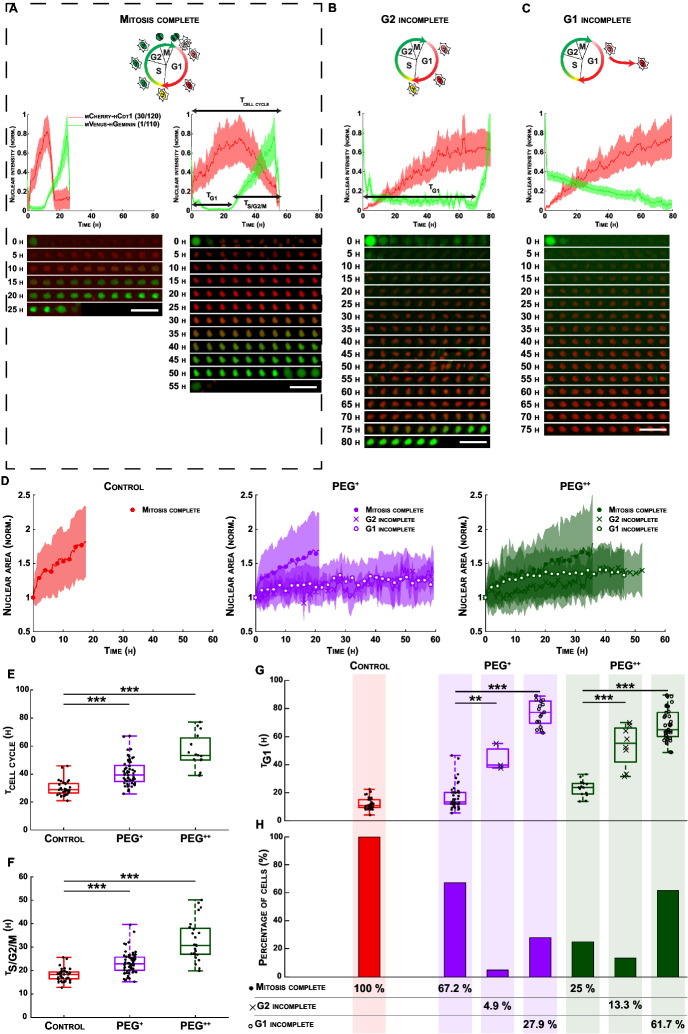
At a single cell level, increase in osmotic pressure leads to the emergence of distinct cell subpopulations with impaired nuclear growth and delayed or arrested cell cycle. (**A**–**C**) Normalized FUCCI2 fluorescence intensity inside of segmented nuclei (middle panels), corresponding fluorescence images over time (bottom panels) and cartoons (top panels) representing the cell cycle phases in three different cell subpopulation: (**A**) cell cycle with mitosis complete (“Mitosis complete”), (**B**) cell cycle with prolonged time in G1 until the cells start the S/G2/M phase (“G2 incomplete”) and (**C**) the cells remain in G1 for the whole duration of the experiment (“G1 incomplete”). The time 0 h for each single cell corresponds to the first frame after division of the parent cell. Scale bars are 100 µm. (**D**) Normalized nuclear area as a function of time for the three experimental groups and the three different cell subpopulations. The line is the average and the shaded area is the standard deviation. (**E**) Duration of the whole cell cycle (time from division to division) for the cell subpopulation “Mitosis complete” (N = 34, 57 and 15 cells for the control (red), PEG^+^ (violet) and PEG^++^ (green) groups). (**F**) Duration of the S/G2/M phase for the cell subpopulation “Mitosis complete” (N = 39, 61 and 24 cells for the control, PEG^+^ and PEG^++^ groups, respectively). (**G**) Duration of the G1 phase in the three different cell subpopulations: “Mitosis complete”, “G2 incomplete” and “G1 incomplete”, for the control, PEG^+^, PEG^++^, groups, respectively. (**H**) Fraction of cells in the three different cell subpopulations, for the groups: control, PEG^+^ and PEG^++^. The plots represent the median, 1st and 3rd quartiles and extrema. Statistical analysis with respect to the control using a two-tailed Wilcoxon rank sum test, n.s: *p* > 0.05, *: *p* < 0.05, **: *p* < 0.01 and ***: *p* < 0.001. The FUCCI2 cartoons were adapted from Sakaue-Sawano et al.^22^, Copyright (2008), with permission from Elsevier.

The original Article has been corrected.

