# Supplementary material for: Osmotic pressure modulates single cell cycle dynamics inducing reversible growth arrest and reactivation of human metastatic cells
Source: Sci Rep. 2021 Jun 29;11:13455. doi: 10.1038/s41598-021-92054-w (PMC8242012; doi:10.1038/s41598-021-92054-w)
Supplement: Supplementary file 1 — Supplementary Information 1. [file 41598_2021_92054_MOESM1_ESM.pdf]

Supplementary Information

Osmotic pressure modulates single cell cycle dynamics inducing reversible growth arrest and reactivation of human metastatic cells

Hubert M. Taïeb, Daniela S. Garske, Jörg Contzen, Manfred Gossen, Luca Bertinetti, Tom Robinson, Amaia Cipitria

Corresponding author: Dr. Amaia Cipitria, amaia.cipitria@mpikg.mpg.de

This PDF file includes:

Supplementary information

Supplementary Figures S1 to S5

Supplementary Movies S1 to S5

Supplementary information

Characterization MDA-FUCCI2 vs. MDA-MB-231 parental cell line

The generated MDA-FUCCI2 cell line was compared with the MDA-MB-231 parental cell line regarding cell attachment, migration and proliferation. For the cell migration assay, two-chambered silicone cell culture inserts (Ibidi, #80209, area of 0.22 cm^2 per chamber) were placed in a 24 well plate (Thermo Fischer, #142475). Both cell types were seeded with a density of 50 000 cells in a total volume of 70 µL media. Cells were allowed to adhere for 5 h before the silicone chamber was removed to create a reproducible; cell-free area of 500 ± 50 µm. Non-adherent cells were washed away by rinsing once with media. Afterwards cells were incubated in 2 mL media and the gap closure was monitored for 20 h using an inverted microscope (Zeiss AxioObserver 7, 10x objective) with a live cell imaging system (Okolab, UNO-T-H-CO2). The cell-free gap area was quantified in percent of the initial cell-free gap area using MATLAB. To assess cell proliferation kinetics, 38 000 cells in 2 mL media were seeded in 24 well plates and the number of cells at days 1, 2, 3 and 4 were quantified using a cell counter (Scepter™ 2.0, Millipore). Finally, to determine cell attachment, the same Ibidi inserts were used. Both cell types were seeded with a density of 2 200 cells in 70 µL and imaged directly after seeding using the same inverted microscope for a duration of 4 hours. A custom-made MATLAB code was used to count round cells, corresponding to suspended cells before they

adhere. No significant differences were found between the generated MDA-FUCCI2 and the MDA-MB-231 parental cell line with regard to cell attachment, migration and proliferation.

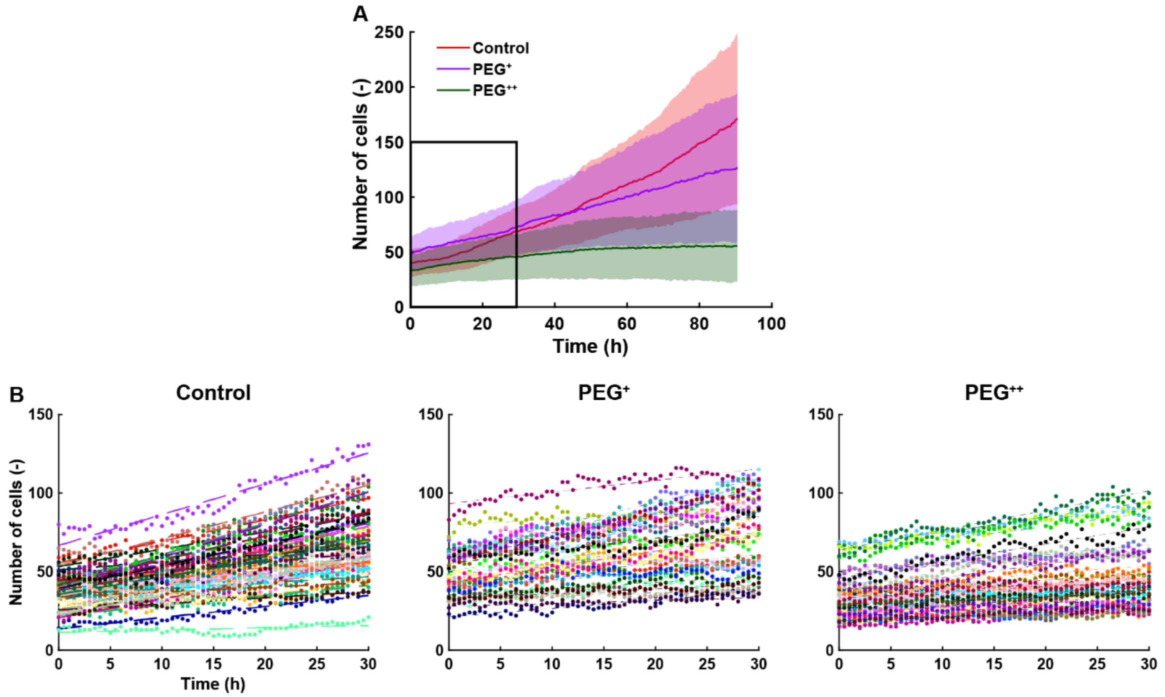

Figure S1. Number of cells as a function of time and explanation for the calculation of the proliferation rate. (**A**) Average and standard deviation of the cell number as a function of time among all repeats (N = 43, 30 and 43 wells for the control, PEG^{+} and PEG^{++} groups respectively). (**B**) Details of each curve per well as indicated by the box in panel A. These curves are used to compute a linear fit (y = ax + b) from 0 to 30 hours, and the slopes correspond to the data in Fig. 1C defined as proliferation rate.

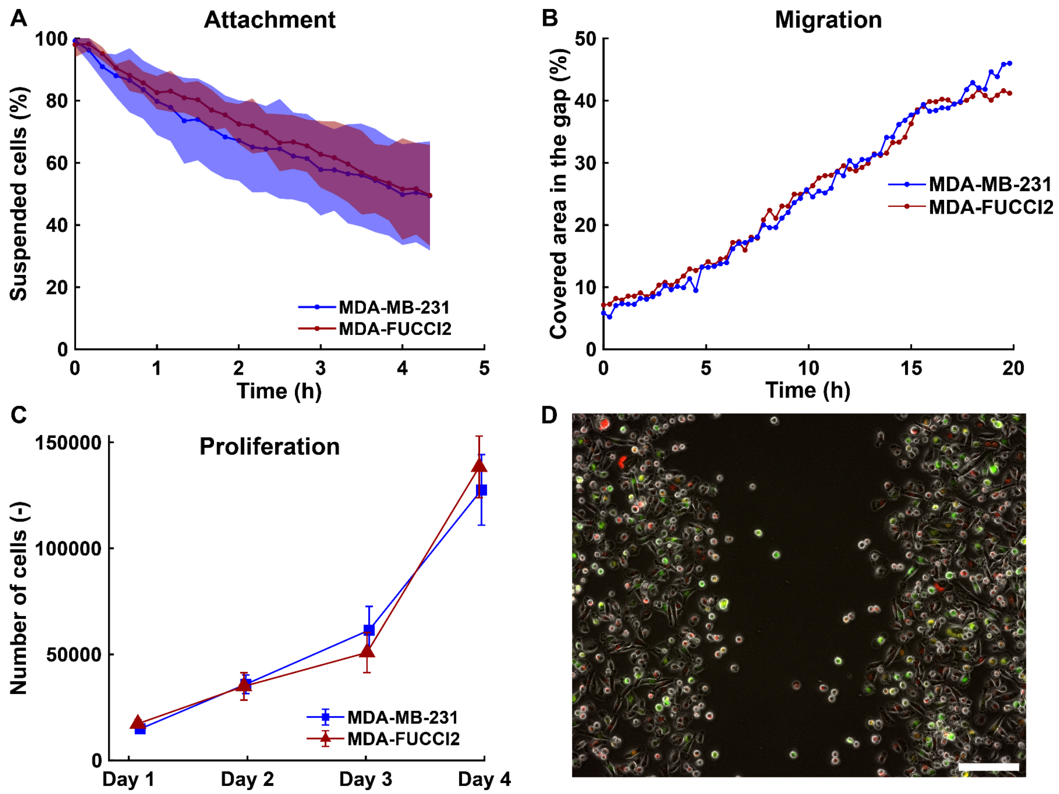

Figure S2. Characterization of MDA-FUCCI2 vs. MDA-MB-231 parental cell line. (**A**) Attachment assay where cells were seeded in a closed compartment and imaged for 4 hours every 30 minutes (N = 4 wells). Initially (t = 0 h), all cells were round and in suspension and, with time, the number of suspended cells decreased as they attached and spread on the bottom of the well. (**B**) Wound healing assay showing the coverage of the gap created by the Ibidi inserts as a function of time. (**C**) Proliferation assay, representing the number of cells as a function of time, measured with a cell counter (N = 6 wells for each group). (**D**) Fluorescent image representing the beginning of the migration assay (t = 0 h), scale bar is 200 µm. Data in **A** and **C** are mean ± standard deviation.

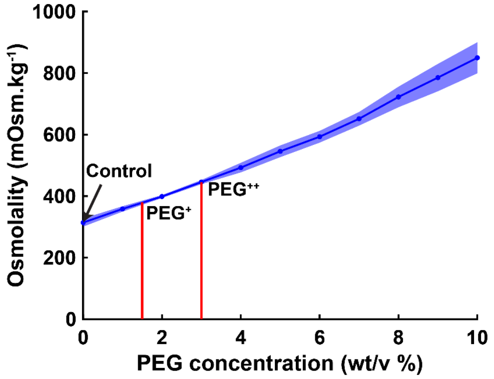

Figure S3. Osmolality as a function of PEG 300 concentration in cell culture media (N = 3). Normal cell culture media has an osmolality of 320 mOsm.kg^{-1} (control). Two hyperosmotic pressures were used in this study: 380 mOsm.kg^{-1} (1.5 wt/v %, PEG^{+}) and 460 mOsm.kg^{-1} (3 wt/v %, PEG^{++}). The curve represents the mean and the shaded area is the standard deviation.

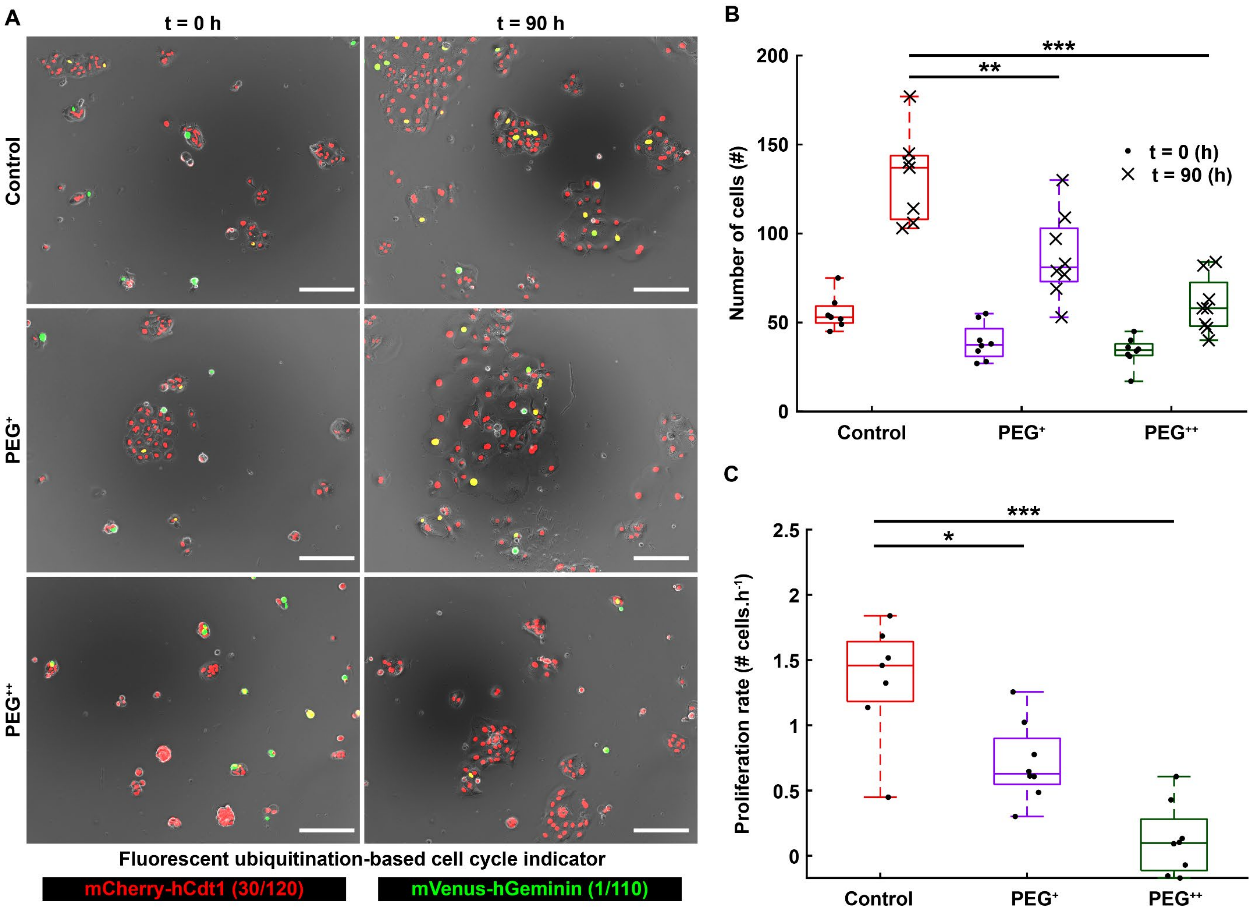

Figure S4. Increase of osmotic pressure slows down the cell cycle dynamics in a concentration-dependent fashion. (**A**) Time lapse of MCF7 FUCCI2 cells exposed to three different osmolalities: 320 mOsm.kg^{-1} (control), 380 mOsm.kg^{-1} (PEG^{+}) and 460 mOsm.kg^{-1} (PEG^{++}). Scale bars are 200 µm. (**B**) Number of cells at time 0 and after 90 hours of imaging (1 biological repeat and N = 7, 8 and 8 number of wells for the control, PEG^{+} and PEG^{++} groups, respectively). (**C**) Proliferation rate are taken from the slope of the curves indicating the number of cells as a function of time, in the range between 0 and 30 hours. The plots represent the median, 1st and 3rd quartiles and extrema. Statistical analysis with respect to the control using a two tailed Wilcoxon rank sum test, n.s: $p > 0.05$, *: $p < 0.05$, **: $p < 0.01$ and ***: $p < 0.001$.

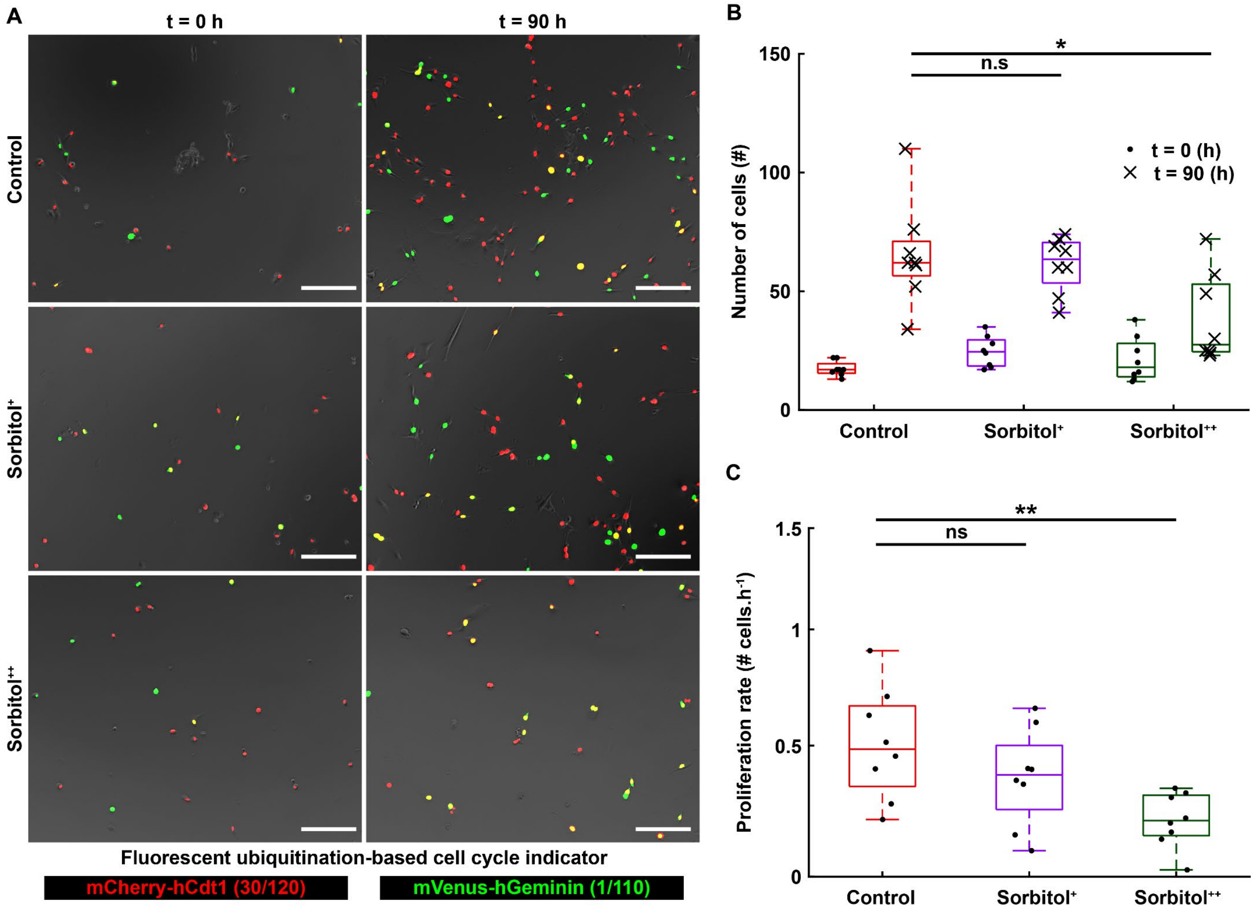

Figure S5. Increase of osmotic pressure via sorbitol slows down the cell cycle dynamics. (**A**) Time lapse of MDA-F2 cells exposed to three different osmolalities: 320 mOsm.kg^{-1} (control), 380 mOsm.kg^{-1} (Sorbitol^{+}) and 460 mOsm.kg^{-1} (Sorbitol^{++}). Scale bars are 200 µm. (**B**) Number of cells at time 0 and after 90 hours of imaging (1 biological repeat and N = 8, 8 and 8 number of wells for the control, Sorbitol^{+} and Sorbitol^{++} groups, respectively). (**C**) Proliferation rate are taken from the slopes of the curves indicating the number of cells as a function of time, in the range between 0 and 30 hours. The plots represent the median, 1st and 3rd quartiles and extrema. Statistical analysis with respect to the control using a two tailed Wilcoxon rank sum test, n.s: $p > 0.05$, *: $p < 0.05$, **: $p < 0.01$ and ***: $p < 0.001$.

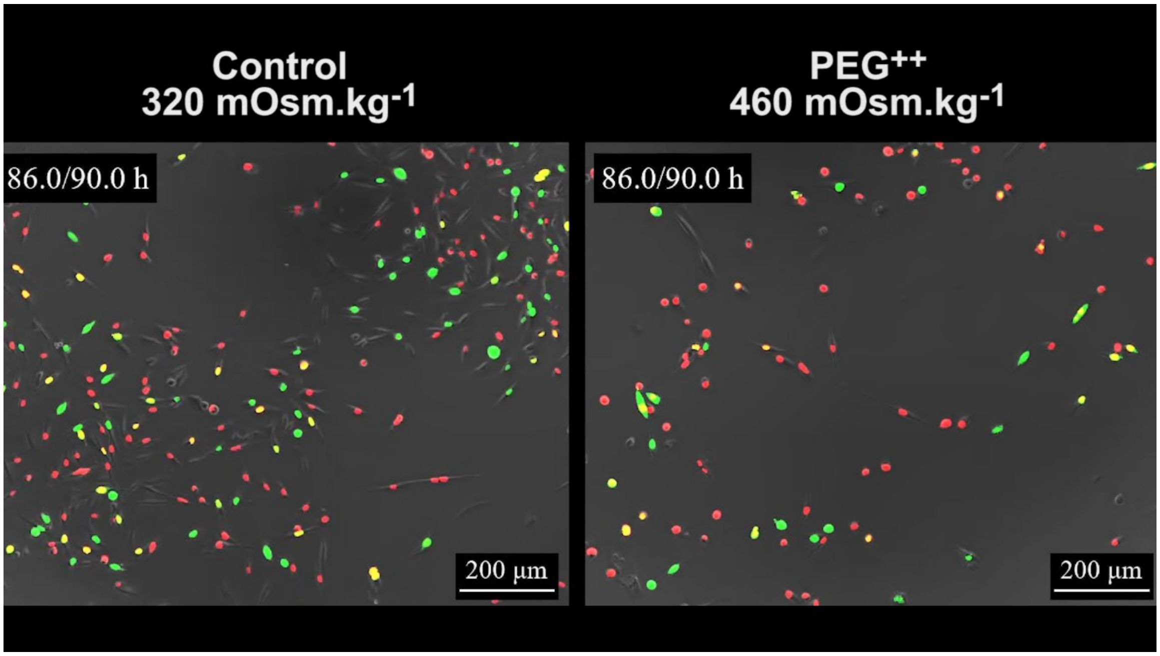

Movie S1 (separate file). Hyperosmotic stress slows down cell cycle dynamics of highly proliferative human metastatic cells. Phase contrast and fluorescence time-lapse imaging of MDA-FUCCI2 cell proliferation in normal (Control) and high hyperosmotic pressure (PEG^{++}). Time in hours.

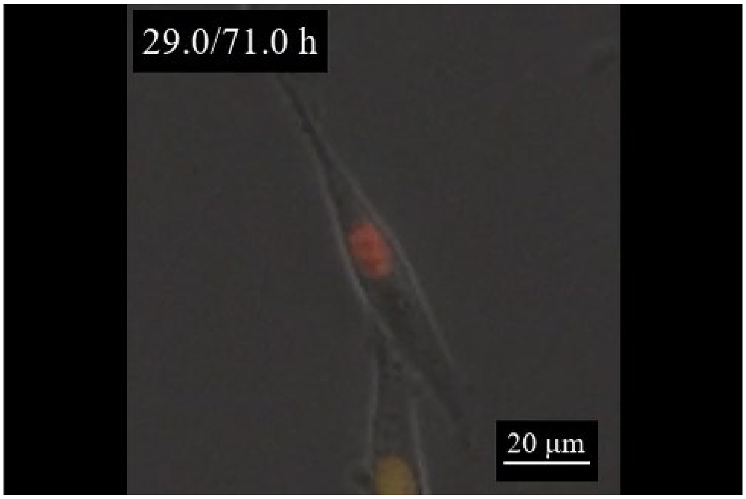

Movie S2 (separate file). Single cell tracking of an osmotically-driven growth arrested MDA-FUCCI2. Phase contrast and fluorescence time-lapse imaging of a single MDA-FUCCI2 cell belonging to the cell subpopulation “G1 incomplete”. Time in hours.

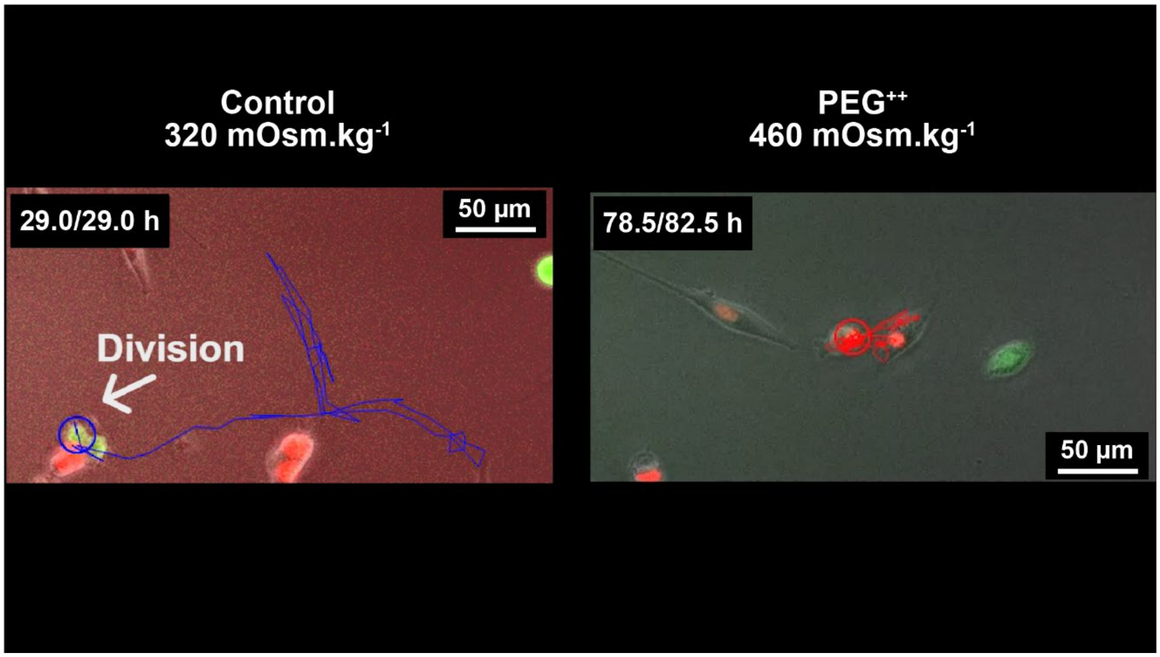

Movie S3 (separate file). Cell migration is slowed down but not arrested under hyperosmotic stress. Phase contrast and fluorescence time-lapse imaging of single MDA-FUCCI2 cells in the control and PEG^{++} group, with an overlay of their trajectory in blue and red, respectively. Time in hours.

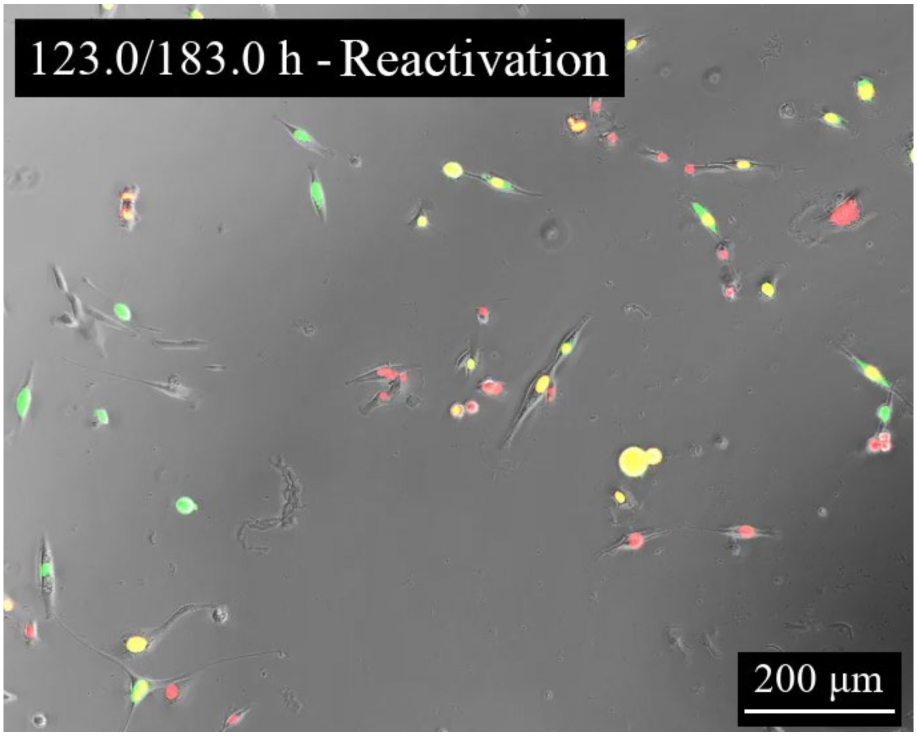

Movie S4 (separate file). Releasing the osmotic pressure resumes cell cycle dynamics of highly proliferative human metastatic cells. Phase contrast and fluorescence time-lapse imaging of MDA-FUCCI2 cell proliferation in the PEG^{+} group, before (0 to 90 hours) and after (90 to 180 hours) reactivation. Time in hours.

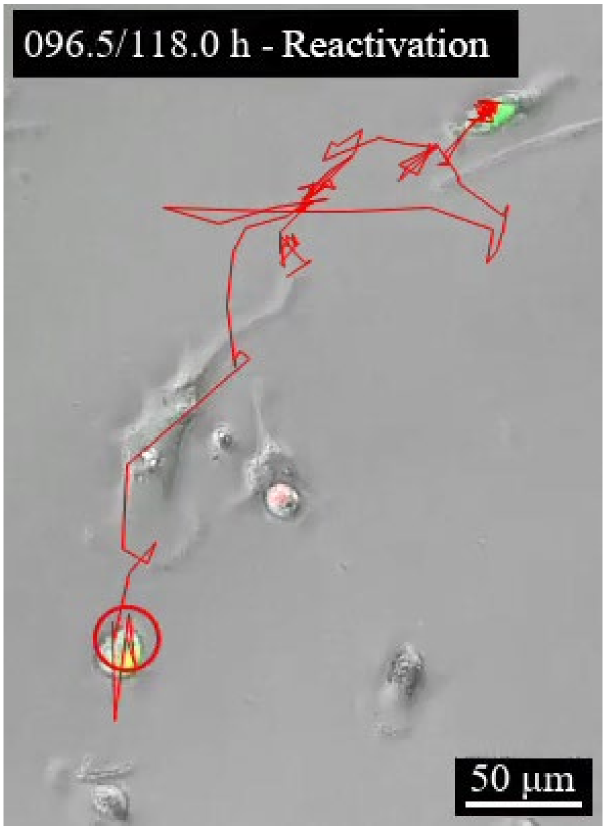

Movie S5 (separate file). Releasing the osmotic pressure resumes the previously impaired cell migration and cell cycle dynamics. Phase contrast and fluorescence time-lapse imaging of a single MDA-FUCCI2 cell, under PEG^{+} condition during the first 90 hours. Upon reactivation, cell migration is recovered as shown by the trajectory in red. Time in hours.
